# Supplementary material for: Alkali-Induced Hydrolysis Facilitates the Encapsulation of Curcumin by Fish (Cyprinus carpio L.) Scale Gelatin
Source: Foods. 2025 Mar 28;14(7):1183. doi: 10.3390/foods14071183 (PMC11989123; doi:10.3390/foods14071183)
Supplement: Supplementary file 1 [file foods-14-01183-s001.zip › foods-3525495-supplementary.pdf]

Supporting Information

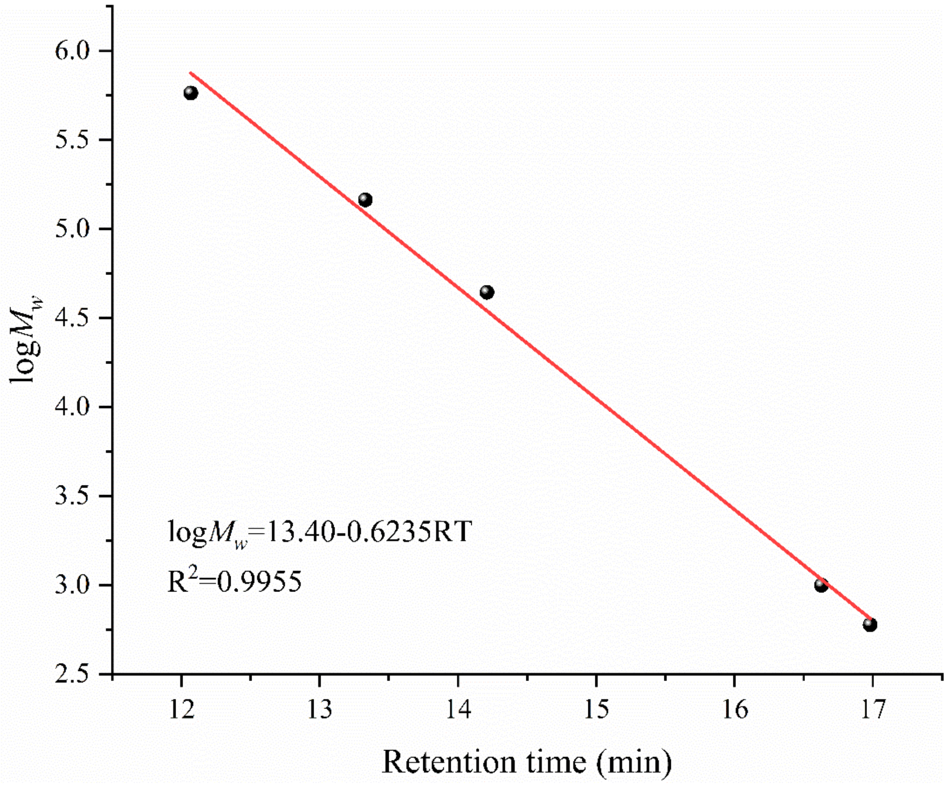

Figure S1 HPLC calibration curve of a series of glucan standards.

Table S1: Secondary structure content (%) of FSG and AFSGs ($AFSG_{2h}$, duration of alkali hydrolysis=2 h; $AFSG_{8h}$, duration of alkali hydrolysis=8 h).

	α-helix (%)	β-sheet (%)	β-turn (%)	Random coil (%)
FSG	12.8±0.2	29±0.2	23.6±0.3	34.6±0.7
$AFSG_{2h}$	3.2±0.1	43.6±0.4	21.2±0.1	32±0.4
$AFSG_{8h}$	3.5±0.1	42.4±0.6	21.7±0.3	32.4±0.2
